# A Rare Case of Metastatic Glioblastoma Diagnosed by Endobronchial Ultrasound-Transbronchial Needle Aspiration

**DOI:** 10.1155/2022/5453420

**Published:** 2022-06-18

**Authors:** Mais Al-Sardi, Ali Alfayez, Yazeed Alwelaie, Abdullah Al-Twairqi, Faris Hamadi, Khalid AlOkla

**Affiliations:** King Fahad Medical City, Riyadh, Saudi Arabia

## Abstract

Glioblastoma is a common primary brain tumor that has a high mortality rate. Reports of intrathoracic metastases are uncommon, with the most commonly reported site for metastases are the lung and pleura. However, involvement of the mediastinum is not well documented, and few reports of confirmed mediastinal metastases diagnosed by endobronchial ultrasound-transbronchial needle aspiration (EBUS-TBNA) exist. Herein, we report a rare case of metastatic glioblastoma to the thorax. A lady in her 40s has been previously diagnosed with intracranial glioblastoma with multiple incidences of disease recurrence despite treatment with chemoradiotherapy, adjuvant chemotherapy, and repeated surgical resections. She presented with dyspnea and pleural effusion, for which radiological imaging revealed lung, pleural, and mediastinal lesions. Further diagnostic workup with EBUS and pleural fluid sampling confirmed metastatic disease to both sites. The pleural fluid showed highly atypical cells positive for GFAP, and EBUS-TBNA immunostains were GFAP, S100, and synaptophysin positive, giving an overall picture consistent with metastatic glioblastoma. The patient was referred for palliative care, and unfortunately, she passed away after several months.

## 1. Introduction

Glioblastoma is a known highly aggressive primary brain tumor. It is the most common primary malignant brain tumor reported in the United States in 2013-2017, constituting 14.5% of all brain tumors, and it carries a very poor prognosis with a 5-year mortality rate of 5.8% [1]. Similarly, in Saudi Arabia Eastern Province, one study reported glioblastoma as the most common pathological type between 2010 and 2015 in all age groups, representing 32% of all neuroepithelial brain tumors [2]. In a 10-year single-center study at King Fahad Medical City (KFMC) in the central region of Saudi Arabia, glioblastoma was similarly reported as the most common primary malignant tumor, constituting 25.6% of all primary brain tumors in the adult population (>18 years) [3]. The median overall survival (OS) for patients treated with a combined modality was 19.7 months [3]. Another retrospective analysis from the same center in Saudi Arabia found that the 6-month progression-free survival (PFS) rate was 43% for all patients and 55% for the combined modality group. As for median OS, it was 13.7 months [4].

Extraneural metastatic disease of glioblastomas is uncommon, with a reported incidence of 0.4-0.5% of the cases [5]. The frequency of incidence reported for extracranial metastatic (ECM) disease has increased over the last decades [6]. The site of metastasis of glioblastoma impacted survival prognosis, and lung metastases were reported to have the worst prognosis [5]. The most commonly reported initial metastases sites are the lung and pleura, followed by the lymph nodes (LNs) [7]. In a more recent meta-analysis, bone metastasis was most common (24.3%), followed by lung and LN metastases, 21.7% and 12.2%, respectively [8]. LN metastasis is more frequently reported to involve the cervical area (62%), followed by mediastinal or hilar LN (32%) [7].

Endobronchial ultrasound-transbronchial needle aspiration (EBUS-TBNA) is a minimally invasive diagnostic modality, most utilized in mediastinal staging and lung cancer diagnosis. However, it can be used as a first-line diagnostic modality in diagnosing mediastinal lymphadenopathy of unknown etiology. Here, we report one case of metastatic, recurrent glioblastoma, diagnosed by LN sampling of the mediastinum using EBUS-TBNA.

## 2. Case Report

Our patient is a 43-year-old lady diagnosed with a right parasagittal brain glioblastoma five years ago. The glioblastoma was WHO (World Health Organization) grade 4 with an unmethylated MGMT (methylguanine-DNA methyltransferase) mutation. She underwent gross resection in July 2016 and received concurrent chemoradiotherapy, which was completed in October 2016. In addition, she received adjuvant TMZ (temozolomide) and high-dose atorvastatin as per KFMC ART study protocol, completed in March 2017 [9]. Unfortunately, her glioblastoma recurred in February of 2018, for which she underwent reresection and received bevacizumab for five months. Despite these interventions, she developed a third recurrence and required resection in November of 2018, and received bevacizumab and irinotecan for six months. In September 2019, she had a fourth recurrence, which was resected, but she had rapid disease progression and was treated with surgical resection and 35 Gy in 10 fractions of radiation therapy. In December 2019, CT scans showed a fifth recurrence of her disease, which was resected again and treated with reirradiation 35 Gy in 10 fractions until February 2020, when her disease progressed again, and she received bevacizumab and lomustine until August 2020. However, her disease progressed again. She had another surgical resection in September 2020 with reirradiation with 35 Gy in 10 fractions in November 2020. She was maintained on bevacizumab and dose dense temozolomide until May 2021, when she complained of shortness of breath.

The patient was referred to our emergency room for admission. She gave a two-week history of shortness of breath associated with productive cough and right-sided pleuritic chest pain. Before coming to the clinic with her new symptoms, she sought medical advice in a nearby clinic and was given a course of antibiotics with no significant improvement in her condition. Her symptoms progressed to the extent that she had severe dyspnea at rest, could not lie flat, and her cough was worse with blood-tinged sputum. She lost her appetite and had a 6-kilogram unintentional weight loss over two months. She did not have any history of fever or exposure history of note. Subsequently, she presented to our emergency department and was found tachypneic, and her other hemodynamics were normal. The patient looked fatigued, and her physical examination was significant for decreased air entry over the right side on chest auscultation with a dull percussion note in the right lower zone.

**Figure 1:**
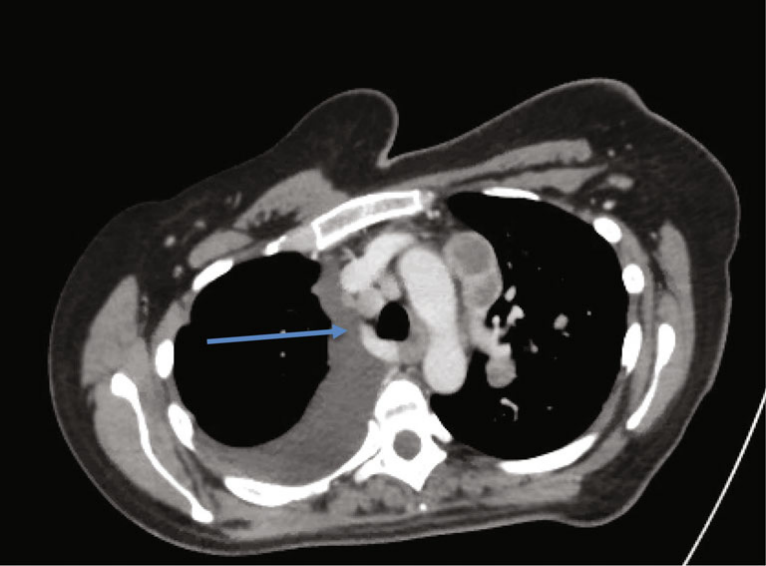
CT chest showing involvement of the right lower paratracheal lymphnodes (Station 4R).

**Figure 2:**
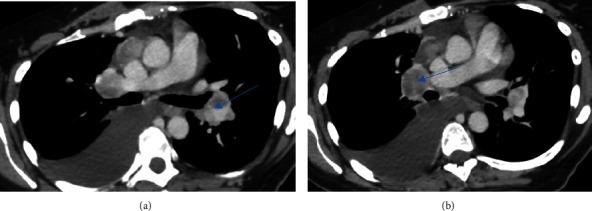
CT chest. (a) Confluent prevascular nodal mass measuring 4.1 × 2.4 cm. (b) Right hilar lymph node measuring 2.5 × 3.1 cm.

Her laboratory investigations showed a WBC (white cell count) of 5.7 × 10^3^/*μ*L and two negative COVID-PCR swabs. Her chest computed tomography showed multiple enlarged necrotic mediastinal and bilateral hilar LN, the largest measuring 4.1 × 2.4 cm. She had a moderate right-sided pleural effusion with multiple bilateral pulmonary nodules, with the largest one in the left lower lobe, measuring 2.4 cm. Multiple lytic lesions in the dorsal vertebrae and a sclerotic focus in the manubrium sternum were identified (Figures 1 and 2).

Bedside thoracentesis was done, draining around 1.2 L of clear yellow fluid initially. The pleural fluid cell count showed 723 × 10^3^/UL WBC with a lymphocytic predominance (78% lymphocytes). Gram stain showed no acid-fast bacilli, and the polymerase chain reaction for mycobacterium DNA was not detected. Pleural fluid cytology showed highly atypical cells, and after sometime, GFAP (glial fibrillary acidic protein) immune staining was positive in some atypical cells. After pleural fluid drainage, the patient's symptoms improved, and she was able to lie flat.

**Figure 3:**
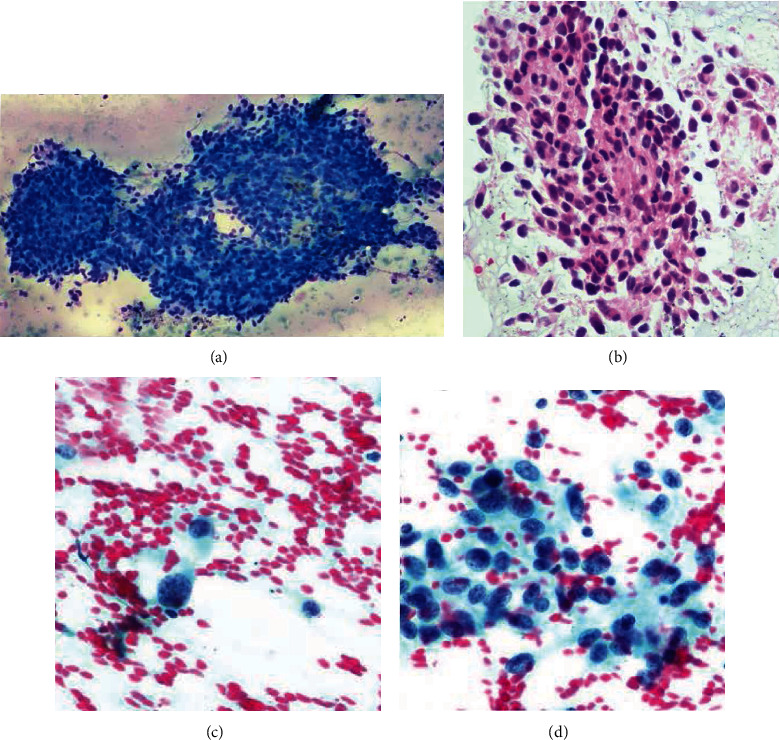
Diff-Quik smear shows clusters of cohesive neoplastic, epithelioid cells with a small amount of cytoplasm (a). The alcoholol-fixed smears demonstrate individual and clustered neoplastic cells with coarse chromatin, ill-defined borders, and nuclear grooves (c, d). The corresponding cellblock material is shown in (b).

**Figure 4:**
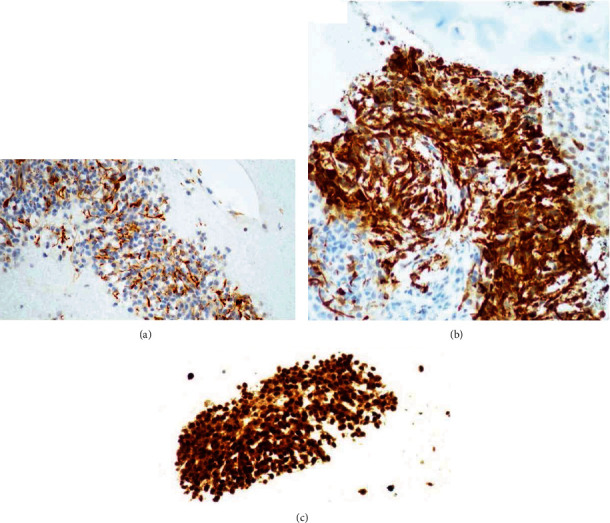
GFAP is immunoreactive in the tumor cells (a). (b) Strong positivity for synaptophysin. S100 protein immunostain demonstrates strong and diffuse positivity (c). Keratin is negative (not shown).

The patient underwent EBUS-TBNA the following day as this case was time-sensitive and to confirm wether she has LN metastasis (which is very rare as mentioned) vs. another primary malignancy or other cause. Five and six needle passes were performed from the right lower paratracheal and right interlobar LNs, respectively. The rapid onsite examination from both stations was consistent with adequate lymph node tissue aquisition and highly suspicious for malignancy, and the samples were sent for further pathological examination and cultures. Cytological smears and cellblock demonstrate clusters of hyperchromatic neoplastic cells with small nucleoli and oval to elongated nuclei in a necrotic background. Cytoplasmic extensions were appreciated in some areas (Figure 3). The tumor cells were negative for broad-spectrum epithelial markers (a panel of keratins was employed). All site-specific epithelial markers were negative. Tumor cells, however, were immunoreactive for GFAP, synaptophysin, and S100 protein (Figure 4). Due to the patient's prior history and immunohistochemical results, the findings were considered consistent with metastatis from the patient's known glioblastoma.

**Figure 5:**
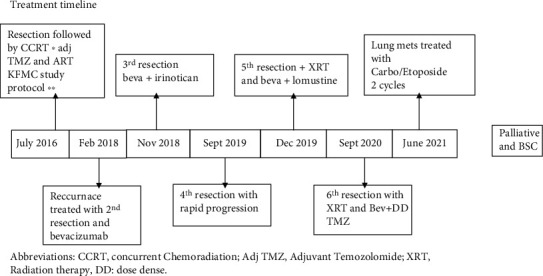
Treatment timeline. Abbreviations: CCRT: concurrent chemoradiation; Adj TMZ: adjuvant temozolomide; XRT: radiation therapy; DD: dose dense.

A trial of carboplatin/etoposide was entertained, and the patient received 2 cycles before her extraneural disease progressed. Next-generation sequencing (NGS) was performed on the tissue samples, but no actionable mutations were found. Her performance status declined, and she was transferred under the care of the palliative team in July 2021. Unfortunately, she passed away after several months. Treatment timeline is shown in Figure 5.

## 3. Discussion

Glioblastoma metastatic disease is known to occur mainly through blood and cerebrospinal fluid (CSF) [10]. Another important metastatic route is the lymphatic system; it was initially thought that the cerebrum was devoid of a lymphatic system and spread to LNs occurs through the cervical lymphatic plexus and the cranial perineural spaces [7]. However, new evidence reports the existence of a functioning lymphatic drainage system [11]. Surgical interventions such as shunt placement have been reported to cause metastases [7, 12]. Metastatic glioblastoma generally occurs after craniotomy, but it has been reported in cases without surgical intervention [7, 13]. The higher incidence following surgery is thought to be coincidental rather than metastatogenic [14].

The diagnostic modalities of metastatic intrathoracic glioblastoma depend on the pattern of disease involvement. Intrathoracic involvement can present as lung nodules, lung masses, pleural effusion, mediastinal masses, and mediastinal lymphadenopathy [15–21]. Similar to our case, the utilization of the cell block technique of pleural fluid in the diagnosis of ECM Glioblastoma was previously reported [22]. Bronchoscopic biopsy has also been previously utilized in the diagnosis of metastatic glioblastoma [20, 23, 24]. Endobronchial fine-needle aspiration (FNA) biopsy has been reported in the diagnosis of glioblastoma from a metastatic lung mass [25]. For suspected mediastinal involvement, endoscopic ultrasonography-guided fine-needle aspiration (EUS-FNA) was reported diagnostic of gliosarcoma, a variant of glioblastoma, from a posterior mediastinal mass [26]. In one study of a metastatic glioblastoma to the lung with mediastinal lymphadenopathy, EBUS-TBNA was employed in the sampling of a subcarinal LN, which was nondiagnostic; however, although the patient underwent mediastinoscopy, it was unclear if the sampled LN was positive for glioblastoma (i.e., false-negative TBNA) [27]. The diagnosis by FNA sampling of cervical LN was previously reported [28–33]. To our knowledge, no reports of EBUS-TBNA diagnostic for glioblastoma from mediastinal LN exist.

In conclusion, ECM of glioblastoma incidence is increasing. Minimally invasive, cost-effective diagnostic modalities of FNA for suspected ECM of glioblastoma have been utilized. This case showed that EBUS-TBNA could rapidly and safely diagnose rare ECM glioblastoma mediastinal LN metastases.

## Data Availability

No data were used to support this study.

## Abbreviations

EBUS-TBNA: Endobronchial ultrasound-transbronchial needle aspiration
KFMC: King Fahad Medical City
LN: Lymph nodes
OS: Overall survival
ECM: Extracranial metastases
WBC: White blood cell
TMZ: Temozolomide
GFAP: Glial fibrillary acidic protein
FNA: Fine-needle aspiration
CCRT: Concurrent chemoradiation
Adj TMZ: Adjuvant temozolomide
XRT: Radiation therapy
DD: Dose dense
